# Considerations on D-mannose Mechanism of Action and Consequent Classification of Marketed Healthcare Products

**DOI:** 10.3389/fphar.2021.636377

**Published:** 2021-03-02

**Authors:** Francesco Scaglione, Umberto M. Musazzi, Paola Minghetti

**Affiliations:** ^1^ Department of Oncology and Hemato-Oncology, Università Degli Studi Di Milano, Milan, Italy; ^2^ Clinical Pharmacology Unit, ASST Grande Ospedale Metropolitano Niguarda, Milan, Italy; ^3^ Department of Pharmaceutical Sciences, Università Degli Studi Di Milano, Milan, Italy

**Keywords:** D-mannose, urinary tract infection, FimH adhesin, medical device, product administrative classification

## Abstract

Urinary tract infections (UTIs) are very common disorders that affect adult women. Indeed, 50% of all women suffer from UTIs at least one time in their lifetime; 20–40% of them experience recurrent episodes. The majority of UTIs seems to be due to uropathogenic *Escherichia coli* that invades urothelial cells and forms quiescent bacterial reservoirs. Recurrences of UTIs are often treated with non-prescribed antibiotics by the patients, with increased issues connected to antibiotics resistance. D-mannose, a monosaccharide that is absorbed but not metabolized by the human body, has been proposed as an alternative approach for managing UTIs since it can inhibit the bacterial adhesion to the urothelium. This manuscript discusses the mechanisms through which D-mannose acts to highlight the regulatory aspects relevant for determining the administrative category of healthcare products placed on the market. The existing literature permits to conclude that the anti-adhesive effect of D-mannose cannot be considered as a pharmacological effect and, therefore, D-mannose-based products should be classified as medical devices composed of substances.

## Introduction

Urinary tract infections (UTIs) are very common disorders affecting adult women. In the United States, 11% of women report at least one UTI event per year (Hickling and Nitti, 2013). Half of the adult women have at least one UTI episode during their lifetime, with 20–40% experiencing recurrent episodes (Kyriakides et al., 2020). Their incidence is between 150 and 250 million cases worldwide per year.

The latest guidelines of European Association of Urology (EAU) on urological infections define UTIs as “recurrences of uncomplicated and/or complicated UTIs, with a frequency of at least three UTIs/year or two UTIs in the last six months” (Bonkat et al., 2020; Kyriakides et al., 2020). The economic burden is relevant: annual direct and indirect costs of UTIs for overall patients are estimated to be from $2.4 billion to $3.5 billion in the United States, based on the source (Foxman, 2010; Eells et al., 2014; Lenger et al., 2020). Moreover, the patients’ work productivity, personal and family responsibilities, quality of life, and sexual well-being are affected by UTIs (Ciani et al., 2013; Renard et al., 2014; Lenger et al., 2020).

The majority of UTIs seems to be due to uropathogenic *Escherichia coli* (UPEC), which is able to colonize urothelium, invade epithelial cells and form quiescent biofilms (Sarshar et al., 2020). This bacterial reservoir may provide a source for bacterial persistence and UTIs recurrence (Berry et al., 2009). Besides, recurrent UTIs may be also caused by the periodic translocation of other bacteria which originate from the gastrointestinal tract or reinfections due to external sources (e.g., sexual activity, and catheterization) (Thänert et al., 2019).

Recurrences of UTIs are often treated with antibiotics. Indeed, UTIs are frequently managed with non-prescribed antibiotics in the EU (European Commission, 2017a). Considering the inexorable rise of antimicrobial resistance worldwide, great efforts have been made by the European Commission to reduce the improper and free use of antibiotics by patients (European Commission, 2017b). In this context, there is a need for alternative approaches, which can reduce the consumption of antibiotics counteracting the emergence of resistance (Loubet et al., 2020).

A promising therapeutic approach is the use of anti-adhesive agents, like D-mannose (Kyriakides et al., 2020; Lenger et al., 2020; Loubet et al., 2020; Sarshar et al., 2020). It is an inert monosaccharide that is excreted not metabolized in urine after oral absorption and inhibits the bacterial adhesion to the urothelium (Wellens et al., 2008).

The purpose of this manuscript is to analyze the mechanisms through which D-mannose acts to prevent UPEC colonization of the urinary tract and highlight the regulatory aspects relevant for determining the administrative category of healthcare products placed on the market. D-mannose has been marketed as a medical device in the EU since 2015 for the prevention of UTIs. However, the latest version of the “Manual on borderline and classification in the community regulatory framework for medical devices” (European Commission, 2019) has opened a scientific discussion on the classification of the mechanism of action of D-mannose (i.e., pharmacological or physical). Such aspect is particularly relevant also for the identification of the proper administrative classification of other healthcare products containing therapeutic substances. Indeed, based on their mechanism of action they must fulfill the regulatory framework on medicinal products or on medical devices containing substances to be placed on the market.

## Mechanism of Action of D-Mannose

**FIGURE 1:**
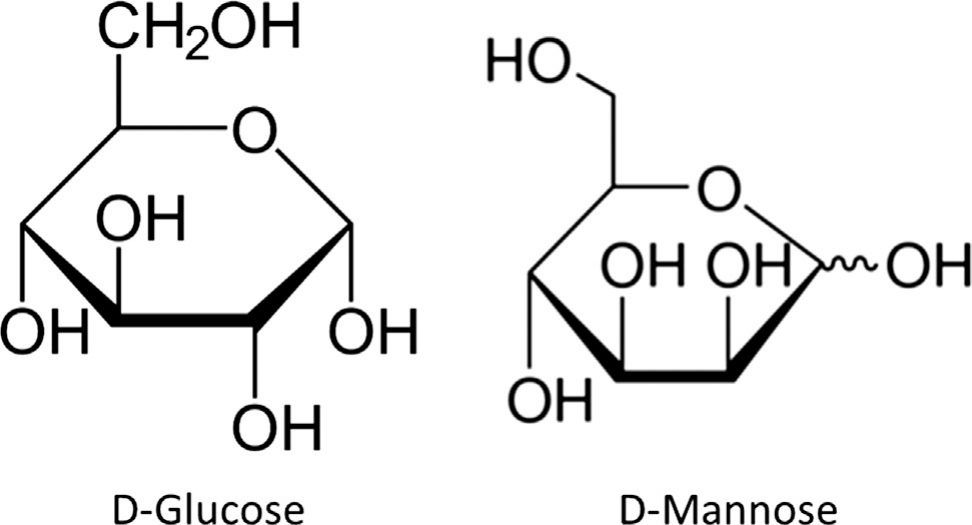
Chemical structure of D-glucose and D-mannose.

D-Mannose is a natural aldohexose sugar differing from glucose by inversion of one of the four chiral centers of the molecule, precisely that on the carbon atom in the second position (Figure 1). This sugar is physiologically present in the human body and it is involved in the immunoregulation (Zhang et al., 2017) and has other important biological roles, such as the glycosylation of many proteins (Alton et al., 1998). However, the D-mannose used in the N-glycosylation and glycerophospholipid anchor synthesis seems to derive from enzymatic stereospecific interconversion of glucose, not from diet intake. Indeed, although D-mannose is a simple sugar, it is not metabolized in humans (Herman, 1971; Alton et al., 1997; Alton et al., 1998; Duran et al., 2004; Sharon, 2006; Sharma et al., 2014). Pharmacokinetic studies have shown that at least 90% of ingested D-mannose is efficiently absorbed in the upper intestine, and rapidly excreted from the bloodstream. Its plasma half-time ranges from 30 min to some hours. The large amount is excreted unconverted into the urine within 30–60 min; the remainder is excreted within the following 8 h (Alton et al., 1998). No significant increase in glucose blood levels occurs during this time, and D-mannose is detectable in the tissues only in trace level.

The rationale to the use of D-mannose in UTIs prophylaxis is based on its competitive inhibition of bacterial adherence to urothelial cells due to its similar structure to the binding site of type 1 fimbriae expressed on the bacteria (Schaeffer et al., 1980; Wellens et al., 2008; Altarac and Papes, 2013). Indeed, UPEC can adhere and, therefore, colonize the urothelium taking advantage from the interaction between type 1 fimbriae and the glycoproteins expressed by epithelial cells (Zhou et al., 2001; Cusumano et al., 2011; Aydin et al., 2015; Kalas et al., 2017). Type 1 fimbriae have a strong affinity for the terminal mannose epitopes of uroplakin Ia (UPIa), a highly mannosylated membrane protein that coats superficial epithelial umbrella cells of the urinary tract (Sauer et al., 2019). A similar adhesion mechanism has been suggested between other types of microorganisms (Pak et al., 2001; Taganna et al., 2011) and host’s tissues. For example, type 1 fimbriae have been documented on other members of the Enterobacteriaceae family, including *Klebsiella pneumoniae*, *Shigella flexneri*, *Salmonella typhimurium*, *Serratia marcescens*, and *Enterobacter cloacae* (Jones et al., 1995; Pan et al., 1997; Lenger et al., 2020). Many of these are also uropathogens of recurrent UTIs. Moreover, it has been demonstrated that fimbriae play a key role also in extraintestinal pathogenic *E. coli* invasion and translocation through the intestinal epithelium (Poole et al., 2017).

D-mannose can bind the FimH adhesin, which is located at the tip of the type 1 fimbria of UPEC and is the virulence factor in UTI pathogenesis (Wellens et al., 2008; Mydock-McGrane et al., 2017). The “coverage” of the binding sites of FimH adhesin by D-mannose occurs through reversible hydrophobic/hydrophilic interactions (e.g., hydrogen bonds, van der Waals forces) without altering the protein conformation (Ohman et al., 1985). D-mannose can establish up to 12 direct hydrogen bonds with main- (Phe1 and Asp47) and sidechains (Asp54, Gl133, Asn135, Asp140) of the FimH adhesin (Feenstra et al., 2017; Mydock-McGrane et al., 2017). However, it is noteworthy that the D-isomer and the α-anomer (α-D-mannose) is mainly responsible for the anti-adhesive effect; modifications in such conformation and/or chemical structure may result in a drop of the binding affinity (Mydock-McGrane et al., 2017). The anti-adhesive effect of other sugars (e.g., glucose, galactose) is significantly lower or negligible (Bouckaert et al., 2005).

However, the anti-adhesive effect of D-mannose is not a consequence of a pharmacological effect on either the host body or the microorganism. It has been demonstrated that, when D-mannose is pre-incubated with human epithelial cells, it does not significantly affect bacteria adhesive capabilities (Schaeffer et al., 1980). Moreover, D-mannose binds the fimbriae, which are not receptors since they are not able to recognize or respond to endogenous chemical signals (Madison et al., 1994). Indeed, any pharmacological action must comprise both a pharmacokinetic and a pharmacodynamic phase, which is related to the so-called “receptor concept” (Flemlee et al., 2012). Although D-mannose shows a concentration-dependent effect, its interaction with the FimH adhesin neither causes nor blocks signal transduction, and a subsequent biochemical reaction (Scribano et al., 2020), which are generally related to the “receptor concept”. On the contrary, the formation of the D-mannose-bacteria complex promotes only the microorganisms’ washout during micturition. Indeed, if urine contains sufficiently high levels of free D-mannose to saturate the FimH adhesin of UPEC, bacteria are unable to grapple onto the epithelial cells and are flushed away by shear forces due to the urinary flow (Ofek et al., 1982; Schwartz et al., 2013). Starting from such scientific evidence, D-mannose and its derivatives (e.g., α-D-mannosides) have been investigated as non-antibiotic prevention strategies for both acute and recurrent UTIs (Kranjcec et al., 2014; Porru et al., 2014; Domenici et al., 2016; Phé et al., 2017; Parrino et al., 2019; Mainini et al., 2020). Moreover, due to this physical mechanism of action, D-mannose has a negligible risk of developing bacterial resistance, unlike antibiotics (Sarshar et al., 2020; Scribano et al., 2020).

## Regulatory Aspects Relevant to Borderline Medical Devices and Medicinal Products

D-mannose is contained both in the EU and the United States in different types of healthcare products, such as food supplements (Altarac and Papes, 2013), and class IIa medical devices. The rational of such healthcare products has its bases on several clinical studies demonstrating the efficacy of D-mannose for the prophylaxis of UTIs (Kranjcec et al., 2014; Porru et al., 2014; Domenici et al., 2016; Phé et al., 2017; Mainini et al., 2020).

The current EU regulatory framework on healthcare products has been created to protect citizens’ health. Therefore, the higher the risks for the consumers, the more restrictive the provisions of the regulatory framework required for placing that product on the market. For example, a product developed for supplementing the normal diet (food supplement) is considered, from a regulatory point of view, less critical than a product developed for a specific therapeutic indication, such as preventing or treating a bacterial infection (i.e., medicinal product or medical device). EU directives and regulations classify the different types of healthcare products based on their intended use. The administrative classification determines the regulatory requirements that a manufacturer/developer must fulfill to place a product on the market. Consequently, the requirements for medicinal products and medical devices are more stringent than those for food supplements, since they are developed with the aim to diagnose, prevent, or treat human diseases. In this context, D-mannose containing products intended to be used for UTIs prophylaxis cannot be marketed as food supplements due to the claimed therapeutic indication, but they fall in the class of medical devices or in that of medicinal products. Here again, although such distinction may appear a minor and technical issue, it has a strong impact on the assessment of the benefit/risk balance of the product because the current regulatory frameworks on medicinal products and medical devices are different.

Medicinal products contain “a substance, or a combination of substances presented as having properties for treating or preventing disease in human beings or which may be used in or administered to human beings either to restore, correct or modify physiological functions by exerting a pharmacological, immunological or metabolic action or to making a medical diagnosis” (European Parliament and Council, 2001). Medical devices, on the other hand, “are instruments, apparatus, appliances, software, implants, reagents, materials, or other articles intended by the manufacturer to be used, alone or in combination, for human beings for medical purposes such as 1) diagnosis, prevention, monitoring, prediction, prognosis, treatment or alleviation of disease; 2) diagnosis, monitoring, treatment, alleviation of, or compensation for, an injury or disability; 3) investigation, replacement or modification of the anatomy or a physiological or pathological process or state; 4) providing information through *in vitro* examination of specimens derived from the human body, including organ, blood and tissue donations; and which does not achieve its principal intended action by pharmacological, immunological or metabolic means, in or on the human body, but which may be assisted in its function by such means.” (European Parliament and Council, 2017).

From both definitions, the medical application of medicinal products and medical devices is evident. Moreover, the EU jurisprudence clarified that, for having a therapeutic activity, a healthcare product should act not only on the human body, but also on microorganisms (e.g., bacteria, virus) inducing a response that has a direct impact on functions of the human being. As an example, in the judgment on the case of chlorhexidine containing mouthwash, the EU Court states that such a product shall not be considered as a cosmetic one since the mechanism of action of chlorhexidine is pharmacological (European Union Court, 2012). Indeed, even if such substance has not a direct pharmacological interaction with a human cellular constituent (e.g., buccal mucosa), its interaction (killing) with the bacteria, viruses, or parasites influences positively on the physiological functions of the constituents of the buccal cavity.

The difference between medicinal products and medical devices resides, therefore, in the mechanism of action. Unlike medicinal products, the therapeutic action of medical devices is not due to a pharmacological, immunological, or metabolic mechanism, but a physical/mechanical means. However, in some cases, a clear distinction between the two is difficult (e.g., medical devices composed of substances). The contact between the substance and a cellular constituent is expected for both products, and therefore a key point for distinguishing them resides into the nature of such a contact. The mechanism of action is pharmacological if such a contact induces a response in the cellular constituents (e.g., activation/block of a cellular pathway), it is physical if the interaction is inert. Indeed, from a scientific point of view, pharmacology can be defined as the study of substances that interact with living systems through chemical processes (Katzung, 2017). Pharmacodynamic interactions usually occur by a substance binding to “regulatory” molecules (i.e., receptors) inducing activation or inhibition of normal body processes. Such concepts have been translated into the current regulatory framework of medical devices. The main guideline on the classification of borderline products defines a pharmacological means as “an interaction between the molecules of the substances in question and a cellular constituent, usually referred to as a receptor, which either results in a direct response, or which block the responses to another agent. Although not a completely reliable criterion, the presence of a dose-response correlation is indicative of a pharmacological effect” (European Parliament and Council, 2001; European Commission, 2015a). Therefore, from both a scientific and a regulatory point of view, a pharmacological action can be established based on three criteria: 1) the existence of an interaction between the molecule and a cellular constituent of an organism, 2) such interaction induces a direct response, activating or inhibiting normal processes in the organism, and 3) the induced response should restore, correct, or modify the physiological functions in the human beings. However, according to the EU jurisprudence, products containing a substance which has a physiological effect cannot be automatically classified as medicinal products “by function” if the pharmacological, immunological, or metabolic effect is not demonstrated based on the established scientific knowledge (European Union Court, 2009; European Union Court, 2012).

Borderline situations, like D-mannose, are particularly frequent in the case of medical devices in which substances are responsible for the therapeutic action, but for which robust scientific evidence on the exact nature of the mechanism of action is lacking. In this context, manuals and guidelines issued from the EU authorities support manufacturers in the correct classification of the healthcare product (European Commission, 2015a; European Commission, 2015b; European Commission, 2019). The case-studies reported in such regulatory guidelines can be grouped in three classes based on the relevant data to determine the nature of their therapeutic action: 1) scientific evidence demonstrates that the mechanism of action is pharmacological, immunological, or metabolic and, therefore, the product must be classified as medicinal product; 2) scientific evidence suggests that the substance may have more than one mechanism of action. For such scenario, the administrative classification of the healthcare product strongly relies on which is the prevalent mechanism of action, and how much ancillary are the others; 3) scientific evidence shows that the mechanism of action is physical/mechanical and, therefore, the product must be classified as a medical device.

Most of the borderline products reported in the manual and guidelines fall in the first class since the pharmacological mechanism of action is clearly demonstrated in the literature (European Commission, 2015b; European Commission, 2019). For example, mustard packs, which are marketed to alleviate the symptoms of common colds and other respiratory diseases, act pharmacologically since their heating and analgesic effect is due to a direct cellular and tissue response (e.g., capillaries expansion and reduced nerve sensitivity) activated by the interaction between the mustard seed components and skin receptors. A classic example of the second scenario is provided by healthcare products containing derivatives of hyaluronic acid. Since 1997, they have been marketed as synovial fluid replacements for viscosupplementation and to restore the physiological and rheological states of the arthritic joint tissue (Richards et al., 2016). Their mechanism of action is mainly mechanical/physical: hyaluronic acid has a similar structure and rheological properties of the components responsible for the viscoelasticity of synovial fluid. However, hyaluronic acid can suppress the production of pro-inflammatory mediators and proteases, the function of immune cells involved in the arthritic process (Carrington Reid, 2013). For low-molecular-weight hylans, this pharmacological action cannot be considered ancillary to the rheological effects on synovial fluids and, therefore, the products should be classified as medicinal products (European Parliament and Council, 2017).

In a few cases, the available evidence supports a physical/mechanical mechanism of action and, therefore, the product can be marketed as a medical device containing substances. As an example, the polysaccharide-resin-honey complex has been marketed as a medical device to treat acute cough because it forms a physical and protective coating on the oral and pharyngeal mucosa (Cohen et al., 2017). Moreover, calcium alginate and oxidized cellulose are hemostatic medical devices since they adhere to platelets’ surface triggering their adhesion and aggregation (European Commission, 2015b).

The case of D-mannose is particularly debated in the scientific community, especially since it was included in the version 1.22 of “Manual on borderline and classification in the community regulatory framework for medical devices.” (European Commission, 2019). It classifies the D-mannose mechanism of action as pharmacological, observing that interactions between such sugar and FimH adhesin can inhibit the adhesion and colonization of UPEC on the urothelium. However, as clearly highlighted by the review of the literature, the nature of the interaction of D-mannose and FimH adhesin does not fulfill with the “pharmacological means” definition of MEDDEV 2.1/3 rev 3 (European Commission, 2015b). On the contrary, literature data suggests that the interaction between D-mannose and FimH adhesin does not produce any activation or inhibition response either to the bacteria or to the epithelial cells of urothelium (Schaeffer et al., 1980; Scribano et al., 2020). The D-mannose inhibition of bacteria adhesion seems, therefore, based on inert physical interactions, which are comparable to those between the platelets and calcium alginate or oxidized cellulose.

## Conclusion

D-mannose can bind type 1 fimbriae of UPEC forming a physical “coating”, which is not harmful to the bacteria, but can prevent their binding to the urothelium and, therefore, the onset of the disease. D-mannose is absorbed, but not metabolized by the human body and it is excreted intact in urine. Neither ancillary effects on bacteria, nor urothelium have been reported in the literature yet. Although the administrative classification of D-mannose-based products has been debated, the anti-adhesive effect of D-mannose cannot be considered as a pharmacological effect: it interacts with bacterial constituents to promote UPEC washing out, but inducing neither a direct and measurable response in the microorganism, nor in the human beings. Consequently, in the EU, healthcare products containing D-mannose indicated for UTI prophylaxis should be classified as a medical device composed of substances.
